# Oropouche Virus: Comparative Study of Two Strains Reveals Distinct Innate Immunity Modulation

**DOI:** 10.3390/v18080828

**Published:** 2026-07-28

**Authors:** Letizia Rizzo, Giulia Alessandri, Gianni Gori Savellini, Sara Caldrer, Concetta Castilletti, Maria Grazia Cusi

**Affiliations:** 1 Department of Medical Biotechnologies, University of Siena, Viale Bracci 16, 53100 Siena, Italy; l.rizzo2@student.unisi.it (L.R.); giulia.alessandri@student.unisi.it (G.A.); gianni.gori@unisi.it (G.G.S.); 2 Virology and Emerging Pathogens Unit, Department of Infectious, Tropical Diseases and Microbiology, IRCCS Sacro Cuore Don Calabria Hospital, Negrar di Valpolicella, 37024 Verona, Italy; sara.caldrer@sacrocuore.it (S.C.); concetta.castilletti@sacrocuore.it (C.C.); 3 Microbiology and Virology Unit, Santa Maria delle Scotte University Hospital, Viale Bracci 16, 53100 Siena, Italy

**Keywords:** Oropouche virus, non-structural NSs protein, interferon beta, RPB1 subunit protein, virus fitness

## Abstract

Oropouche virus (OROV), an Orthobunyavirus of the *Peribunyaviridae* family, is usually transmitted to humans by biting midges, while, to date, cases of human-to-human transmission have not been reported. Although OROV’s clinical manifestation is usually characterized by mild symptoms, cases of Central Nervous System (CNS) infections have been reported. The non-structural protein NSs of many bunyaviruses and phleboviruses acts as a major virulence factor. Previous studies have demonstrated that OROV NSs also behaves as a suppressor of the host IFN-α/β responses. Furthermore, OROV NSs protein promotes cellular RPB1 proteasomal degradation to suppress RNA synthesis. In the present work, we perform a comparative study on two OROV strains (BeAn19991 and IRCCS-SCDC_1/2024) to further characterize the molecular mechanisms by which OROV NSs inhibits IFN-β expression. Our results demonstrate that the NSs protein mediates a reduction in IFN-β promoter activation, hindering RIG-I and IRF-3, therefore acting on multiple steps of the IFN-β signaling pathway. However, this suppression seems to reside in the blockage of RPB1 rather than a direct effect on those mediators. Moreover, we investigated the behavior of the two virus strains in a human glioblastoma cell line derived from brain (DBTRG.05MG cells) and fibroblasts (MRC-5 cells), observing different replication kinetics, degradation activity towards RPB1, and strength/timing of IFN-β modulation.

## 1. Introduction

Oropouche virus (OROV) is a member of the Orthobunyavirus genus within the *Peribunyaviridae* family [1], classified under the Simbu serogroup, which comprises two phylogenetic subclades A and B [2]. In the Americas, three naturally occurring reassortants of OROV have been identified to date: Iquitos virus, Madre de Dios virus, and Perdões virus [3,4]. OROV is a neglected and emerging arbovirus endemic to South and Central America, including Brazil, Colombia, Ecuador, French Guiana, Panama, and Peru, known to infect both humans and animals. In urban transmission cycles, humans are the only known vertebrate hosts, with transmission primarily mediated by biting midges (*Culicoides paraensis*) and mosquitoes (*Culex quinquefasciatus*) [5,6,7]. The most common clinical manifestations of OROV infection are Oropouche fever, a febrile illness characterized by high fever, headache, myalgia, arthralgia, and photophobia [8,9]. Although generally self-limiting, neurological complications such as meningitis and encephalitis have also been reported [10,11]. Moreover, a recent study suggests that OROV infection during pregnancy may lead to adverse outcomes such as fetal death, miscarriage, or microcephaly, resembling those observed following Zika virus (ZIKV) infections [12,13,14,15]. Since its first identification in the Amazon basin in the 20th century, OROV has caused over 30 epidemics and thousands of confirmed infections [16,17,18]. Like many other arboviruses, its geographic distribution is expanding due to climate change, increased human mobility, and urbanization, raising concerns about its potential global public health impact. Notably, in 2024, five imported cases of OROV infection were reported in Italy from travelers returning from Cuba, representing the first documented introduction of OROV into Europe [19,20,21,22]. Given the widespread distribution of *Culicoides* species in Europe, vectors also responsible for the spread of Schmallenberg virus and Bluetongue virus in livestock, OROV poses a potential threat to both human and veterinary health, underscoring the need for effective environmental surveillance and rapid diagnostic measures, particularly during the summer [23,24,25]. Like other Orthobunyaviruses, OROV possesses a segmented, negative-sense, single-stranded RNA genome comprising three segments: Large (L), Medium (M), and Small (S). These segments encode six proteins, including both structural and non-structural components. The L segment encodes the RNA-dependent RNA polymerase (RdRp), which catalyzes RNA replication and mRNA transcription. The M segment encodes a polyprotein that is post-translationally cleaved into two structural glycoproteins (Gn and Gc) and the non-structural NSm protein. The S segment encodes the N and NSs proteins, with NSs being produced by downstream AUG initiation from the same transcript [26,27]. OROV NSs plays a critical role in viral replication and host immune evasion by suppressing type I interferon (IFN-α/β) responses, as demonstrated by in vitro studies using recombinant OROV lacking the NSs gene [27,28]. Previous studies have shown that recombinant OROV lacking the NSs gene induces robust IFN-β production, whereas wild-type (WT) virus elicits only limited cytokine expression during the early stages of infection [28]. Furthermore, the antagonistic function of OROV NSs has been shown to depend critically on its nine C-terminal amino acids [28]. More recently, Jurado-Cobena et al. further elucidated the mechanism underlying NSs-mediated immune antagonism, demonstrating that NSs suppresses host transcription through nucleolar targeting and RNA polymerase II (RNAPII) destabilization via RPB1-mediated degradation [29]. This process reduces the levels of the hyperphosphorylated RPB1 IIo form and consequently causes a global suppression of cellular mRNA synthesis, thereby partially impairing type I IFN signaling downstream of IFNAR activation, representing an evolutionarily acquired immune evasion strategy [29,30].

Among the factors involved in IFN regulation, the transcription factors IRF-3 and IRF-7 play pivotal roles. Previous studies have also suggested that additional factors contribute to the modulation of type I IFN responses during OROV infection, including molecules involved in downstream antiviral signaling, such as TRAF3 (TNF receptor-associated factor 3) and STAT1, as well as host miRNAs, particularly miR-217 and miR-576-3p, which facilitate immune evasion and promote viral replication [31,32,33]. These findings have advanced our understanding of OROV pathogenesis and identified NSs as a promising antiviral target. In this study, we aimed to further characterize the molecular mechanisms by which OROV NSs inhibit IFN-β expression in human cell systems using naturally occurring virus variants rather than recombinant viruses as employed in previous studies [30]. In addition, comparative studies using the prototype BeAn19991 strain and the IRCCS-SCDC_1/2024 reassortant virus, recently imported into Europe from Cuba, will investigate whether different viral fitness or NSs activity could be associated with neuroinvasion. The two NSs protein variants (BeAn19991 and IRCCS-SCDC_1/2024) differ by two amino acid substitutions (I_67_T and S_72_N), which may influence the antagonistic function of NSs, as they are located upstream of the C-terminal region containing a functionally important domain [28].

Therefore, as a preliminary approach, we selected two human-derived cell systems: a human glioblastoma cell line representing a potential target tissue of viral neuroinvasion, and primary fibroblasts, which constitute one of the earliest cellular targets following viral transmission in vivo [34,35,36].

To this end, viral growth kinetics and IFN-β modulation were investigated in these cell systems to provide further insights into the interactions between different OROV strains and the host innate immune response.

## 2. Materials and Methods

### 2.1. Cells, Viruses and Chemicals

Human embryonic kidney Lenti-X 293T cells (Takara Bio, Saint-Germain-en-Laye, France) and Vero E6 (ATCC CRL-1586) were cultured in high glucose (4.5 g/L) Dulbecco’s modified Eagle’s medium (DMEM) (EuroClone, Milan, Italy) supplemented with 100 U/mL penicillin/streptomycin (EuroClone) and 10% heat-inactivated fetal bovine serum (FCS) (EuroClone) at 37 °C. The human glioblastoma cell line DBTRG.05MG cells (Leibniz Institute, Braunschweig, Germany) were cultured in RPMI 1640 medium (EuroClone) supplemented with 100 U/mL penicillin/streptomycin (EuroClone) and 10% heat-inactivated fetal bovine serum (FCS) (EuroClone) at 37 °C. The human lung fibroblast cell line MRC-5 cells (ATCC-CCL-171) were cultured in DMEM (EuroClone, Milan, Italy) supplemented with 100 U/mL penicillin/streptomycin (EuroClone) and 10% heat-inactivated fetal bovine serum (FCS) (EuroClone) at 37 °C. Oropouche virus (OROV), strain IRCCS-SCDC_1/2024 (GenBank Acc. N° PQ363070.1) was isolated on Vero E6 cells at the Department of Infectious—Tropical Diseases and Microbiology, IRCCS Sacro Cuore Don Calabria Hospital (Negrar di Valpolicella, Italy), while the BeAn19991 (GenBank Acc. N° MG747539.1) was kindly provided by Professor Jan Felix Drexler (Institute for Virology, Charité—Universitätsmedizin, Berlin, Germany). Working viral seeds were prepared on Vero E6 cells, titrated by micro-method on the same cell line, and stored at −80 °C for further use. Transfections were performed using the jetOPTIMUS Transfection Reagent (Polyplus, Illkirch, France) following the manufacturer’s instructions. The proteasome inhibitor MG-132 was purchased from Merck Millipore (Milan, Italy) and used at 1 μM; actinomycin D (ActD) was purchased from Life Technologies (Milan, Italy) and used at 5 μg/mL final concentration.

### 2.2. Antibodies

Antibodies used in Western blotting were anti-6xHis (Cell Signaling Technology, #2366, Leiden, The Netherlands) anti-actin as a loading control (Life Technologies) and anti-FLAG-M2 mouse monoclonal antibodies (Merck Millipore, #F1804) at 1:1000 dilution, in-house rabbit TOSV_N serum diluted 1:200, anti-IRF3 (Santa Cruz Biotechnology Inc., Heidelberg, Germany, #FL-425) at 1:500 dilution and eGFP monoclonal antibody (eBioscience™-ThermoFisher Scientific, #GF28R, Milan, Italy) at 1:500 dilution in sterile Phosphate-Buffered Saline (PBS). Phospho-RNA pol II (Ser2/Ser5) CTD (RPB1 subunit) polyclonal antibody (Life Technologies, #PA5-17563) was used to evaluate cellular levels of endogenous enzyme. Horseradish peroxidase (HRP)-conjugated anti-Rabbit IgG (H+L) (Promega, Milan, Italy), diluted 1:2500, or (HRP)-conjugated anti-mouse IgG secondary antibody (Merck Millipore), diluted 1:5000, were used as secondary antibodies.

### 2.3. Plasmids

Viral RNA was extracted from Vero E6 supernatant of OROV-infected cells using a QIAamp viral RNA mini kit (Qiagen, Milan, Italy). The NSs (nt 38-313) coding gene was amplified by reverse transcriptase (RT)-polymerase chain reaction (PCR) from purified viral RNA with specific primers (available upon request) (Sigma-Aldrich, Milan, Italy) by using the SuperScript III One-Step RT-PCR with Platinum Taq (Life Technologies, Milan, Italy) by one cycle of reverse transcription at 55 °C for 30 min and 94 °C for 5 min followed by 40 cycles of PCR (1 min at 94 °C; 30 s at 56 °C; 30 s at 68 °C). The amplified gene was cloned into the pcDNA4HisMax-A plasmid (Life Technologies) in frame with the 6xHis tag by standard procedures. All plasmid sequences were verified by Sanger sequencing. The plasmid expressing the Toscana virus (TOSV) strain SI-1812 (GenBank Acc. N° EU327772.1) N gene was prepared by cloning the specific genomic sequence (nt 1070-1831) into the pcDNA3.1(+) plasmid (Life Technologies). The correct sequence of the constructs was confirmed by sequencing (Eurofins Genomics, Milan, Italy). The reporter plasmid encoding Firefly Luciferase downstream of the complete interferon-beta promoter (pINF-β-Luc) and the FLAG-tagged human RIG-I expression plasmid were kindly provided by Prof. A. García-Sastre (Mount Sinai School of Medicine, New York, NY, USA). The plasmids expressing human IRF-3 and its mutant IRF3-5D were kindly provided by L. Martinez-Sobrido (University of Rochester Medical Center School of Medicine and Dentistry, Rochester, NY, USA). The *Renilla* Luciferase pRL-SV40 reporter plasmid was purchased from Promega.

### 2.4. Luciferase Reporter Assay

To study the BeAn19991 or IRCCS-SCDC_1/2024 OROV NSs protein activity on INF-β promoter (pINF-β) activation, a luciferase-based reporter assay was used. 2 × 10^5^ Lenti-X 293T cells were seeded in 24-well plates and, after overnight (o/n) incubation, the cells were transfected. Briefly, 0.2 µg of INF-β-Luc, 0.05 μg of RIG-I or IRF3/IRF3-5D plasmids were co-transfected. Twenty ng of pSV40-RL were co-transfected as an internal control. Where indicated, variable amounts (ranging from 0.1 to 0.5 μg) of BeAn19991 or IRCCS-SCDC_1/2024 OROV NSs-expressing plasmids were co-transfected. An empty plasmid was used to normalize total DNA amount. Twelve or thirty-six hours post-transfection, cells were stimulated with 2 μg/well of poly(I:C) by transfection and collected after an additional 12 h. Luciferase activities were measured on cell sample lysates using the Dual-Luciferase reporter assay reagent (Promega) according to the manufacturer’s instructions. The results are given as the mean fold change in pINF-β-Luc promoter activation ± standard deviation (SD) from at least three independent experiments.

### 2.5. Kinetics of Viral Replication

Monolayers of DBTRG.05MG or MRC-5 cells were seeded at densities of 2 × 10^5^ cells per well in 24-well plates using complete culture medium. The following day, the cultures were infected with a multiplicity of infection (MOI) of 0.1 of the indicated OROV strains and incubated at 37 °C for 1 h. After incubation, the inoculum was carefully removed, and the cells were extensively washed with sterile PBS. Subsequently, 1 mL of the appropriate complete growth medium was added to each well. Cell culture plates were collected at 4, 8, 12, 24, 48, and 72 h post-infection (p.i.) and stored at −80 °C until further analysis. Following thawing and centrifugation, the supernatants containing the virus were collected, and viral titers were determined by microtitration assay on Vero E6 cells seeded in 96-well plates. Briefly, 10-fold dilutions of samples were made in serum-free medium, then 50 μL was plated in a flat-bottom 96-well plate, in quadruplicate. An equal amount of VeroE6 cell suspension (3.8 × 10^5^/mL) was added to each well, and then the plate was incubated at 37 °C for four days. Viral titers were calculated using the Reed–Muench formula and expressed as TCID_50_/mL.

### 2.6. Quantitative Assessment (RT–qPCR) of Gene Expression

Cell pellets from infected DBTRG.05MG or MRC-5, or transfected Lenti-X 293T cells, described above, were subjected to total RNA purification using the RNAeasy PLUS mini kit (Qiagen, Milan, Italy) following the manufacturer’s recommendations. The quality and quantity of the extracted RNA were assessed by spectrophotometry. PCR (RT-qPCR) was used with TaqPath 1-Step Multiplex Master Mix (Applied Biosystems, Milan, Italy) for reverse-transcription quantitative polymerase chain reaction (RT-qPCR) reactions. Pre-cast TaqMan assays (Applied Biosystems) for human IFN-β, RIG-I, and GAPDH were used for specific transcript detection. Toscana virus N gene-specific primer/probe set was described elsewhere [32]. Each sample was run in duplicate on the Applied Biosystems™ 7500 Real-Time PCR System, and the cycle threshold (Ct) values of each gene were normalized against the endogenous GAPDH gene and compared with the negative control. Where indicated, cells were treated with 5 μg/mL of actinomycin D (ActD) or vehicle at 48 h post-transfection and collected at the starting point (T0), 2 h and 5 h post-treatment. Samples were processed for RT-qPCR as described above, and normalization was performed using GAPDH expression. The fold change in specific mRNA content was calculated relative to the mock-infected/transfected samples using the 2^−ΔΔCt^ algorithm. The results are presented as the mean fold change from at least three independent experiments ± standard deviation.

### 2.7. Western Blotting

Whole cell lysates (WCLs) were prepared from transfected Lenti-X 293T cells or infected MRC-5 and DBTRG.05MG cells by using RIPA buffer [50 mM Tris-HCl, 150 mM NaCl, 1 mM EDTA, 1% Triton X-100; pH 7.5] supplemented with anti-protease cocktail (Roche, Milan, Italy) and anti-phosphatase cocktail mix (Pierce, Milan, Italy). Total protein content in the lysates was quantified using the BCA assay (Pierce). A total of 25 µg of protein from each sample was prepared in Laemmli sample buffer, denatured by boiling for 5 min, and then separated by 4–15% MP TGX Stain-Free (Bio-Rad, Milan, Italy) denaturing polyacrylamide gels (SDS-PAGE). The resolved proteins were transferred onto nitrocellulose membranes (Santa Cruz Biotechnology, Heidelberg, Germany) using a Trans-Blot Turbo apparatus (Bio-Rad). After blocking nonspecific membrane sites with 5% non-fat dry milk (in sterile PBS), membranes were probed with specific primary antibodies (as listed above) and specific HRP-conjugated secondary antibodies. The immunoreactive bands were visualized by using the Western blotting Luminol reagent (Santa Cruz Biotechnology), and images were recorded by a ChemiDOC instrument (Bio-Rad). Densitometric analysis was performed by ImageJ software 1.54g. Data were reported as mean fold change ± standard deviation (SD) of normalization target band to the relative loading control band intensities.

### 2.8. Assessment of IFN-β Expression

Medium from DBTRG.05MG and MRC-5 cells, mock-infected or infected as described above, was collected at different time points p.i. and stored in aliquots at −80 °C until used. The amount of secreted IFN-β was assessed by the Enzyme-Linked Immuno-Sorbent Assay (ELISA) VeriKine-HS Human IFN Beta TCM ELISA Kit (PBL Assay Science, Piscataway, NJ, USA), following the manufacturer’s instructions by diluting the sample fivefold in the provided dilution buffer. The results were presented as the mean cytokine concentration (pg/mL) relative to the mock-infected sample ± standard deviation (SD) from at least three independent experiments.

### 2.9. Co-Immunoprecipitation Assay

Lenti-X 293T cells were plated in a 3.5 cm plate and transfected with 2 µg of eGFP-NSs-expressing plasmid or empty plasmid as a control. Samples were collected at 12 h, 24 h, and 48 h post-transfection, and cell pellets were stored at −80 °C until used. For co-immunoprecipitations (Co-IP), WCLs were prepared in 1 mL of RIPA buffer (50 mM TrisHCl [pH 7.5]; 150 mN NaCl; 1 mM EDTA; 1% Triton X-100) supplemented with an anti-protease cocktail (Roche) and anti-phosphatase cocktail mix (Pierce). Lysates were cleared by centrifugation (10 min, 16,000× *g*, 4 °C), and an aliquot (20 μg) was retained as the input control. The remaining samples were incubated overnight at 4 °C with anti-pRPB1 (2 μg) along with 5 μL bed volume of protein G-coupled magnetic beads (Cytiva Italy SRL, Buccinasco, Italy), with gentle rotation. After extensive washes with lysis buffer, bound proteins were eluted by boiling with 2X Laemmli sample buffer, loaded on 4–15% MP TGX Stain-Free (Bio-Rad) denaturing polyacrylamide gels (SDS-PAGE) and probed by immunoblotting, as described above, for both RPB1 and eGFP-NSs presence.

## 3. Results

### 3.1. Oropouche NSs Protein Impairs IFN-β Signaling Pathway

**Figure 1:**
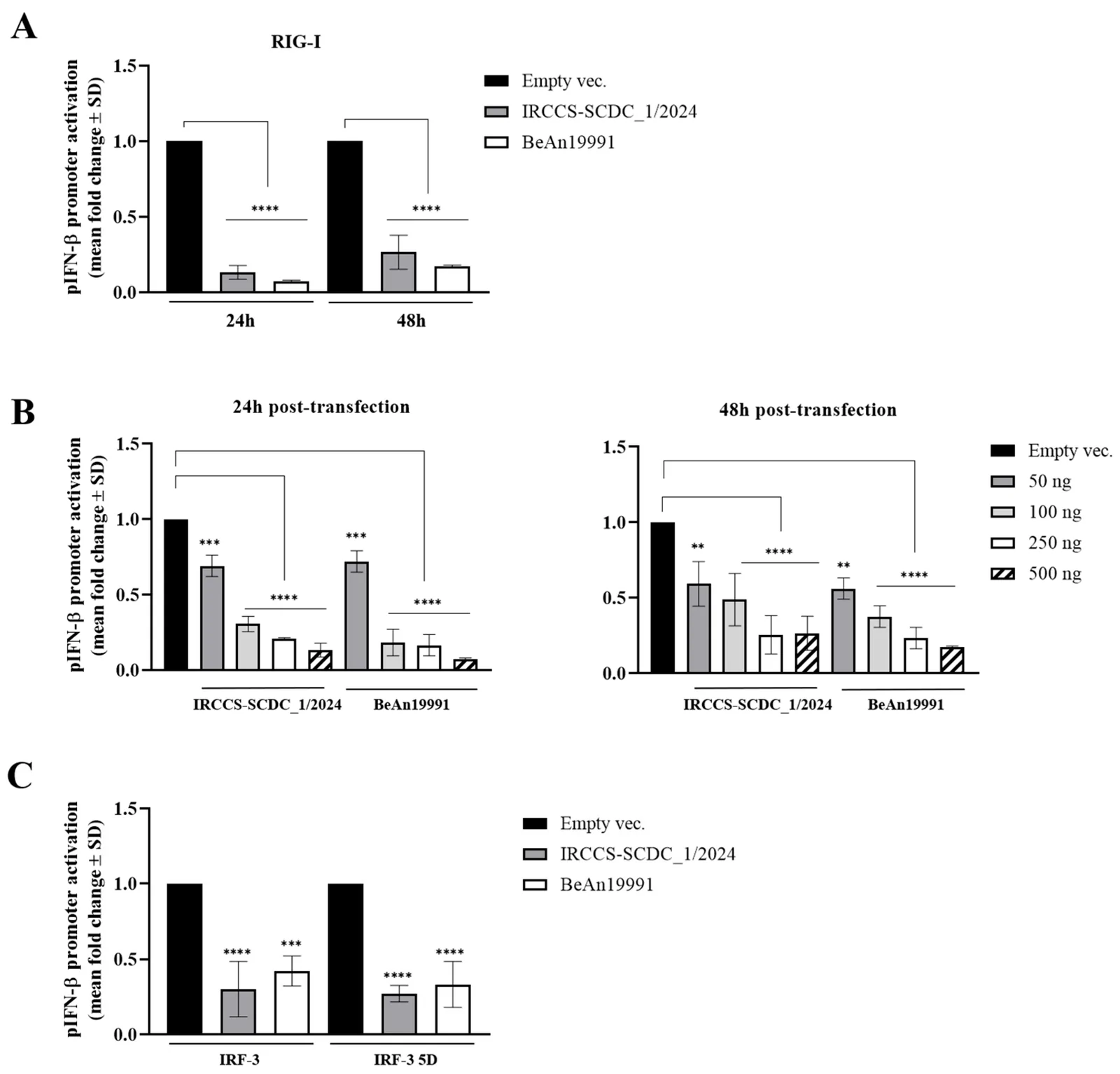
**Inhibitory activity of OROV NSs protein.** (**A**) Lenti-X 293T cells were co-transfected with the IFN-β promoter-driven Firefly luciferase reporter plasmid (pIFN-β), the SV40 promoter-driven *Renilla* luciferase reporter plasmid, and a RIG-I expression plasmid, along with plasmids expressing the BeAn19991 or the IRCCS-SCDC_1/2024 OROV NSs proteins or an empty vector control. At 36 h post-transfection, cells were stimulated by transfection with poly(I:C). Firefly and *Renilla* luciferase activities were measured after an additional 12 h, and fold change was calculated with respect to the empty vector control sample at 24 h or 48 h post-transfection. (**B**) The dose-dependent antagonistic activity of OROV NSs on RIG-I-mediated pIFN-β activation was evaluated at 24 h and 48 h using a luciferase assay. Lenti-X 293T cells were co-transfected with increasing amounts of plasmids expressing the BeAn19991 or the IRCCS-SCDC_1/2024 NSs, together with pIFN-β, RIG-I, and the SV40 promoter-driven *Renilla* luciferase reporter plasmid as an internal control. Where indicated, an empty vector was used as a control. (**C**) Lenti-X 293T cells were co-transfected with plasmids expressing the BeAn19991 or the IRCCS-SCDC_1/2024 OROV NSs, together with reporter plasmids and expression plasmids encoding IRF-3 or its constitutively active form (IRF-3 5D). An empty vector was used in place of NSs as a control. Data are presented as the mean fold induction of pIFN-β activation ± standard deviation (SD) from at least three independent experiments (*n* > 3). Statistical significance was determined by ANOVA and is indicated as follows: **** *p* < 0.0001, *** *p* < 0.001, ** *p* < 0.01. Unless otherwise indicated, differences among samples were not statistically significant.

Previous studies on Oropouche virus (OROV) demonstrated that the viral non-structural protein NSs is able to suppress IFN-β production [28,31]. In the present study, we confirmed that the OROV NSs protein inhibits IFN-β promoter activation and demonstrated that this antagonistic activity targets the upstream signaling component RIG-I within the type I interferon pathway. Furthermore, we performed a comparative analysis of the antagonistic properties of the NSs proteins derived from two OROV strains, IRCCS-SCDC_1/2024 and BeAn19991. The recently circulating variant carries two amino acid substitutions in NSs, T_67_I and N_72_S, compared with BeAn19991. These substitutions may influence the antagonistic activity of the protein, as they are located upstream of the C-terminal functional domain previously implicated in NSs-mediated host antagonism (Figure S1) [28]. Transient expression of the NSs protein from both strains significantly suppressed IFN-β promoter-driven luciferase reporter activity following poly(I:C) stimulation and RIG-I overexpression in Lenti-X 293T cells at 24 h post-transfection (fold change: 0.13 ± 0.04, *p* < 0.0001, and 0.07 ± 0.006, *p* < 0.0001, respectively) (Figure 1A). At 48 h post-transfection, both viral proteins retained their antagonistic activity (fold change: 0.26 ± 0.11, *p* < 0.0001, and 0.17 ± 0.008, *p* < 0.0001, respectively) (Figure 1A).

The specificity of this antagonistic activity was further investigated using a dose-response reporter assay, in which increasing amounts of NSs-expressing plasmids (50–500 ng) were co-transfected with a RIG-I expression plasmid. Reporter activity was assessed at both 24 h and 48 h post-transfection (Figure 1B). A potent inhibitory effect of the NSs proteins from both OROV strains on IFN-β promoter activation was evident as early as 24 h post-transfection with only 50 ng of plasmid DNA, resulting in an approximately 1.5-fold reduction in promoter activity (*p* < 0.003) (Figure 1B). Increasing plasmid concentrations led to a progressive, dose-dependent decrease in reporter gene expression (Figure 1B). However, at 48 h post-transfection, the antagonistic activity of both NSs variants was less pronounced (Figure 1B).

To further characterize the mechanism by which OROV NSs modulates the innate immune response, we evaluated its activity against additional components of the signaling cascade leading to IFN-β induction. We therefore assessed the antagonistic activity of the NSs proteins from both OROV strains on IRF-3-mediated activation of the IFN-β promoter following poly(I:C) stimulation. Both NSs variants significantly suppressed promoter activity, resulting in an approximately 3-fold reduction for IRCCS-SCDC_1/2024 (*p* < 0.0001) and a 2.4-fold reduction for BeAn19991 (*p* = 0.0003) (Figure 1C).

A similar inhibitory effect was observed when a constitutively active IRF-3 mutant (IRF-3-5D) was used. In this experimental setting, poly(I:C) stimulation was not required because of the intrinsic activity of the mutant transcription factor. Nevertheless, co-expression of NSs variants resulted in an approximately 3-fold reduction in IFN-β promoter activation (*p* < 0.0001) (Figure 1C).

Collectively, these findings suggest that OROV NSs interferes with type I interferon signaling at multiple levels, potentially through the broad targeting of key components of the signaling cascade.

### 3.2. OROV NSs Protein Retains Broad-Spectrum Inhibitory Activity on Protein Synthesis

**Figure 2:**
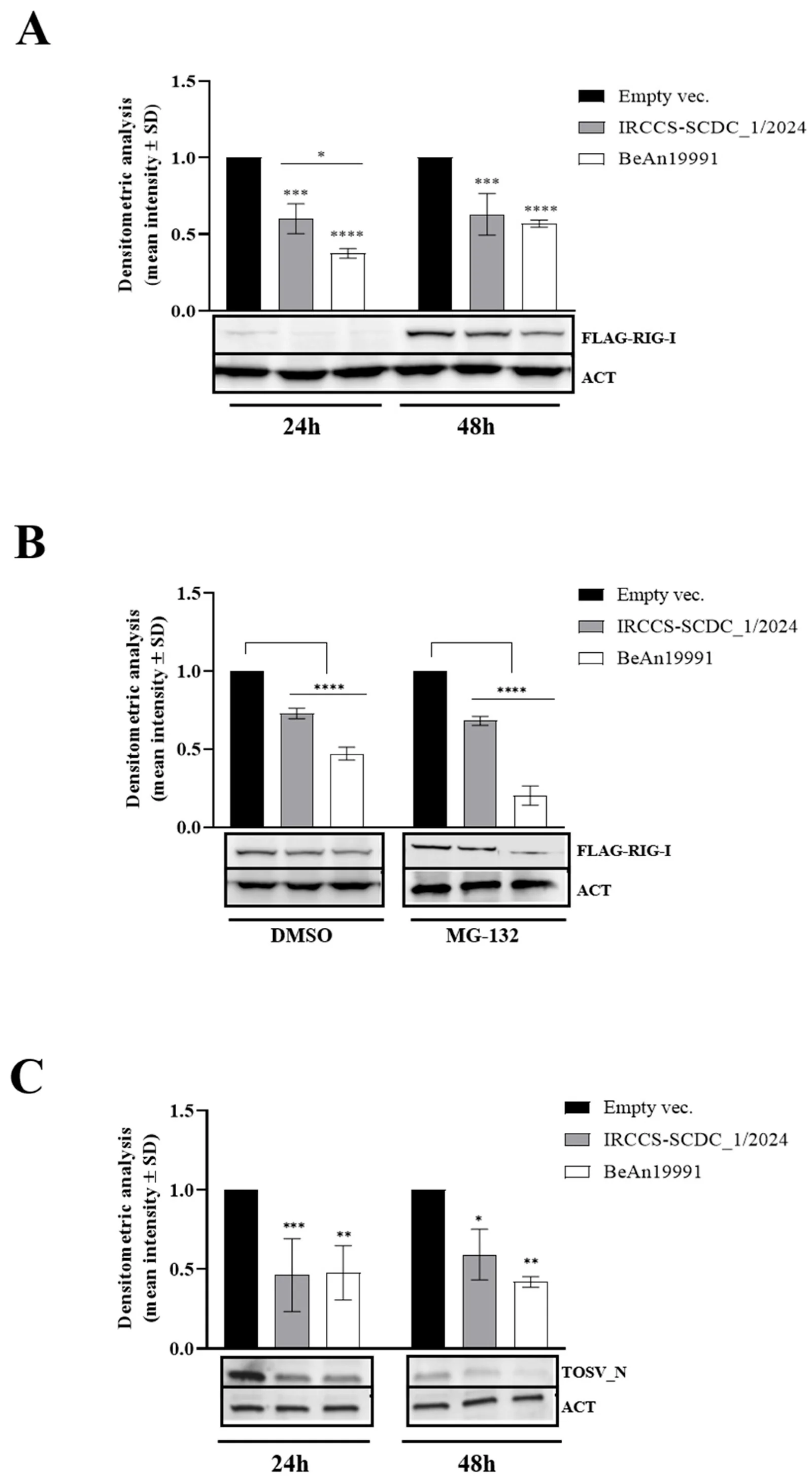
**Evaluation of OROV NSs variants activity towards ectopically expressed proteins.** (**A**) The impact of the OROV NSs protein variants on exogenous RIG-I expression was evaluated in Lenti-X 293T transfected cells by Western blotting. Equal amounts of total whole cell lysates (WCLs) (25 μg) were resolved by SDS-PAGE, and specific antibodies were used to probe for RIG-I (anti-FLAG) and actin (ACT) protein as a loading control at both 24 h and 48 h post-transfection. (**B**) NSs-mediated RIG-I proteasomal degradation was assessed by Western blotting in WCLs of Lenti-X 293T transfected cells either vehicle (DMSO) or proteasome inhibitor MG-132-treated cells collected at 48 h post-transfection. (**C**) The effects of OROV NSs proteins on Toscana virus (TOSV) N protein were similarly determined by Western blotting for N (anti-N) and cellular actin (ACT) as a loading control in WCLs of transfected, vehicle (DMSO) or proteasome inhibitor MG-132-treated cells collected at 48 h post-transfection. A representative image of each Western blot is provided. Densitometric analysis was performed using ImageJ software. Graph values are presented as the mean fold change in RIG-I or TOSV N band intensity ± standard deviations (SDs). Significance was evaluated by ANOVA and reported as * *p* < 0.05, ** *p* < 0.01, *** *p* < 0.001, and **** *p* < 0.0001 between each sample and the control (Empty vector).

It is known that nascent cellular RNA synthesis is impaired in OROV-infected cells [30]. Therefore, we investigated the accumulation of ectopically expressed RIG-I at the protein level in transfected Lenti-X 293T cells by Western blot analysis. At an early time point post-transfection (24 h), a marked reduction in RIG-I protein accumulation was observed in the presence of both NSs proteins, with a 1.7-fold reduction for IRCCS-SCDC_1/2024 (*p* = 0.0002) and a 2.7-fold reduction for BeAn19991 (*p* < 0.0001), as determined by densitometric analysis normalized to the loading control (β-actin) (Figure 2A).

This suppression of RIG-I protein accumulation persisted up to 48 h post-transfection, although to a lesser extent, remaining significantly different from the empty vector control (Figure 2A).

One potential mechanism underlying the activity of OROV NSs could involve ubiquitination and proteasome-mediated degradation of target proteins. To investigate this possibility, we evaluated the effects of the proteasome inhibitor MG-132 on RIG-I accumulation in cells co-expressing NSs proteins. No significant differences were observed between vehicle-treated (DMSO) and MG-132-treated samples (Figure 2B), suggesting that proteasomal degradation is not the primary mechanism employed by OROV NSs to counteract host innate immune responses and reprogram cellular processes.

To determine whether this inhibitory activity was directed against specific targets or reflected a broader effect on protein expression, we assessed the accumulation of an exogenous protein unrelated to innate immune signaling, the nucleoprotein of Toscana virus (TOSV N). As expected, TOSV N accumulation was significantly impaired in the presence of both OROV NSs proteins (*p* ≤ 0.02), resulting in a reduction of more than 50% compared with control conditions (Figure 2C).

**Figure 3:**
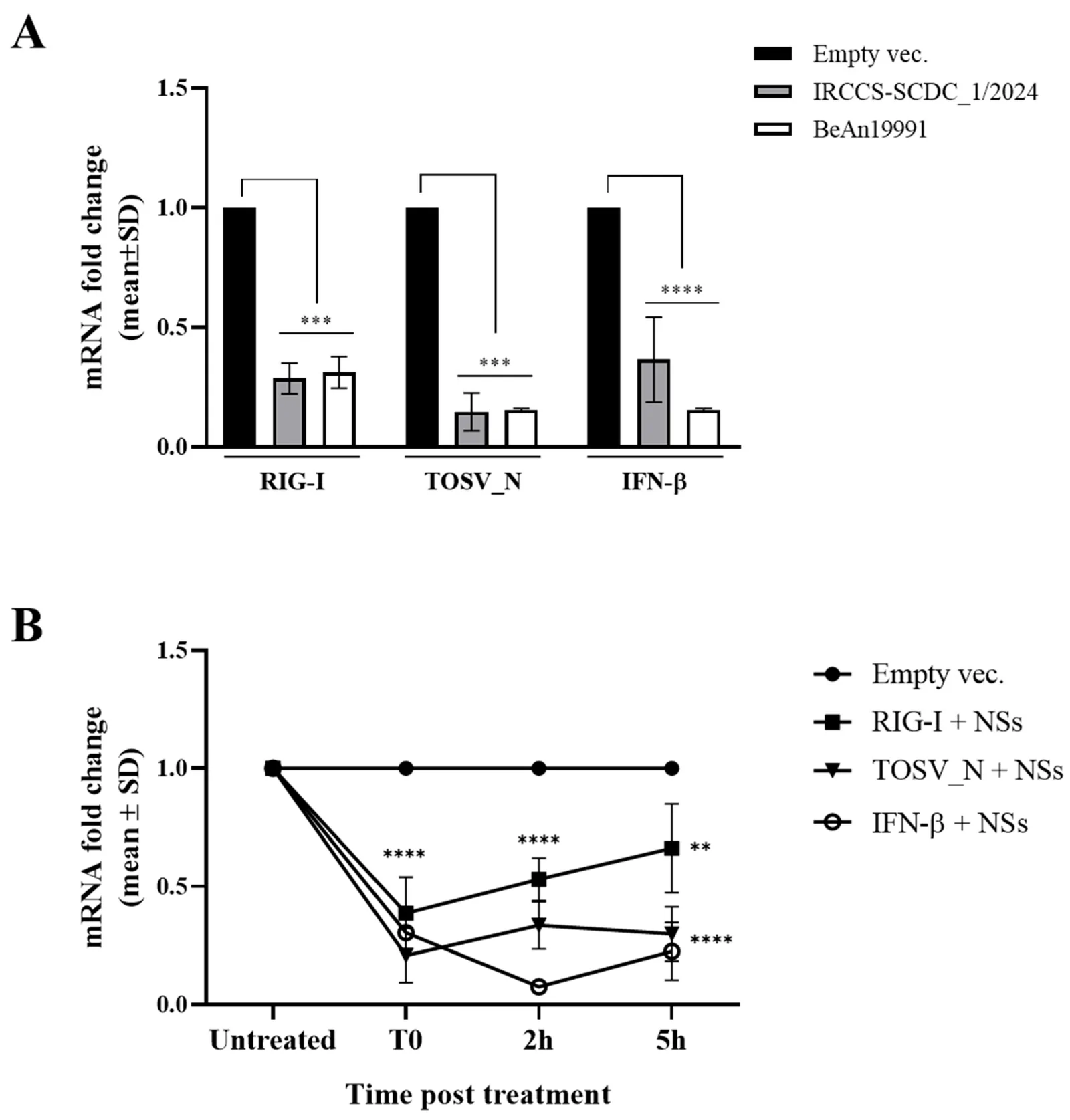
**Evaluation of NSs modulation of different target gene expression.** (**A**) The impact of BeAn19991 or IRCCS-SCDC_1/2024 OROV NSs protein on exogenously expressed RIG-I and TOSV_N or endogenous IFN-β expression was evaluated in Lenti-X 293T transfected cells. Cells were collected at 48 h post-transfection, and total RNA was purified. RIG-I, TOSV_N, IFN-β, and GAPDH mRNAs were quantified by RT-qPCR using the 2^−ΔΔCt^ analysis. Data were presented as mean fold change values ± standard deviations (SD) from three (*n* = 3) different experiments. (**B**) The influence of both BeAn19991 and IRCCS-SCDC_1/2024 OROV NSs protein on cellular or foreign mRNA decay was investigated in Lenti-X 293T cells. A time-course experiment was conducted using actinomycin D (ActD), a transcriptional inhibitor. Cells were collected prior to ActD supplementation (T0) and at indicated time points; after that, total RNA was isolated and assessed for RIG-I, TOSV_N, and endogenous IFN-β mRNA quantification by RT-qPCR using the 2^−ΔΔCt^ analysis. Significance was evaluated by ANOVA and reported as ** *p* < 0.01, *** *p* < 0.001, and **** *p* < 0.0001 between each sample and the control (Empty vector).

To further investigate the molecular basis of this effect, quantitative RT-PCR (RT-qPCR) analyses targeting RIG-I, endogenous IFN-β, and TOSV N transcripts were performed on total RNA extracted from transfected cells at 48 h post-transfection. As shown in Figure 3A, expression of both NSs proteins induced a pronounced transcriptional repression. Indeed, NSs promoted a broad and non-specific transcriptional shutdown, affecting both endogenous and ectopically expressed genes. At 48 h post-transfection, significant reductions in mRNA levels were observed for RIG-I (approximately 3-fold reduction, *p* < 0.0001), TOSV N (approximately 6-fold reduction, *p* < 0.0001), and endogenous IFN-β (3- to 6-fold reduction, *p* < 0.0001), regardless of the NSs proteins expressed (Figure 3A).

To determine whether the observed reduction in transcript abundance resulted from altered mRNA stability rather than transcriptional inhibition, we performed actinomycin D (ActD) chase experiments, a widely used approach for assessing mRNA turnover. Since no substantial differences in this activity were detected between the two NSs proteins, subsequent experiments were carried out using the IRCCS-SCDC_1/2024 NS construct as a representative model.

As shown in Figure 3B, the stability of the analyzed transcripts was not significantly affected by the presence of IRCCS-SCDC_1/2024 NSs over the examined time course (up to 5 h). Although a marked reduction in the basal expression levels of all target mRNAs was already evident at the initial time point (T0) in NSs-expressing cells (*p* < 0.0001), ActD treatment did not significantly alter the decay kinetics of RIG-I, endogenous IFN-β, or TOSV N transcripts compared with control cells.

Taken together, these findings indicate that the suppression of protein expression observed in the presence of OROV NSs is unlikely to be mediated by enhanced mRNA degradation. Rather, the data support a mechanism involving transcriptional repression, consistent with the broad host shutoff activity previously attributed to OROV NSs.

### 3.3. NSs Activity Is Mediated by Its Interaction with RPB1

**Figure 4:**
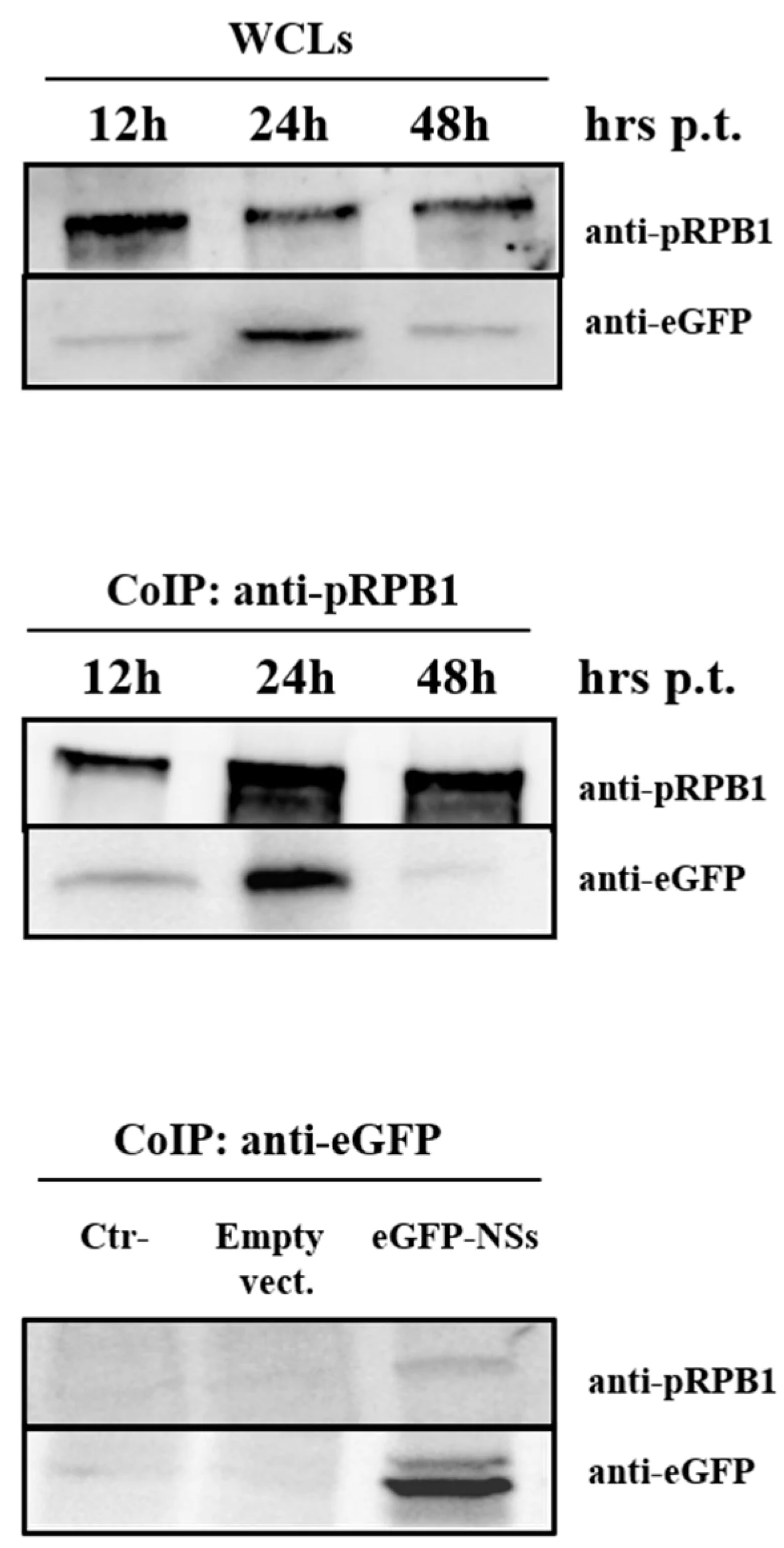
**OROV NSs interact with cellular RPB1 RN polymerase II subunit**. Lenti-X 293T cells were transfected with the BeAn19991 eGFP-tagged NSs construct (eGFP-NSs). Cells were harvested at 12, 24, and 48 h post-transfection, and clarified whole-cell lysates (WCLs) were subjected to co-immunoprecipitation (Co-IP) using an anti-RPB1 antibody and protein G-coupled magnetic beads. The presence of OROV NSs in the immunocomplexes was detected by Western blotting with an anti-eGFP antibody (middle panel). Input levels of eGFP-NSs and cellular RPB1 were assessed by Western blotting using 25 μg of WCLs collected prior to Co-IP (upper panel). To confirm the interaction, untransfected Lenti-X 293T cells and cells transfected with either the empty vector or eGFP-NSs were harvested at 24 h post-transfection. Clarified WCLs were subjected to Co-IP using an anti-eGFP monoclonal antibody, and the interaction with RPB1 was evaluated by Western blotting with the indicated antibodies (lower panel).

To determine whether the inhibitory activity of NSs was associated with a direct interaction with RPB1, co-transfection experiments were performed using eGFP-tagged NSs expression vectors. Co-immunoprecipitation (Co-IP) followed by Western blot analysis demonstrated that OROV NSs directly interact with the cellular RNA polymerase II large subunit RPB1 (Figure 4).

Notably, the interaction was detectable as early as 12 h post-transfection and persisted up to 24 h (Figure 4, middle panel). At 48 h post-transfection, the interaction was no longer observed, likely reflecting the reduced accumulation of the eGFP-NSs protein (Figure 4, upper panel). The interaction was further validated by Co-IP using an anti-eGFP antibody in untransfected Lenti-X 293T cells and in cells transfected with either the empty vector or eGFP-NSs. Although the assay showed lower sensitivity, likely due to the properties of the anti-eGFP antibody, RPB1 was detected exclusively in eGFP-NSs immunoprecipitates and was absent from both control samples. These findings confirm the specific interaction between NSs and RPB1 and exclude any non-specific binding (Figure 4, lower panel). Unfortunately, it was not possible to confirm this interaction during OROV infection because NSs expression levels were too low for reliable detection and no specific antibody against NSs was available. Nevertheless, these findings provide further insight into the molecular mechanisms employed by Orthobunyaviruses to induce host cellular shutoff.

### 3.4. Oropouche Virus Infection Affects RNA Polymerase II Stability

The largest subunit of cellular RNA polymerase II (RPB1) is targeted by several viruses, including OROV, to evade innate immune responses and induce host transcriptional shutoff through proteasome-mediated degradation, thereby preventing cellular mRNA synthesis [29,37,38,39,40]. We therefore investigated whether infection with the two OROV strains differentially affected RPB1 stability.

Preliminary experiments were conducted to determine the most appropriate infectious dose (multiplicity of infection ranging from 0.01 to 1) that would allow efficient and detectable viral replication without causing excessive cell damage that could interfere with the experimental results. Therefore, human glioblastoma DBTRG.05MG cells were infected with either the OROV BeAn19991 or IRCCS-SCDC_1/2024 strain at an MOI of 0.1. Whole cell lysates were collected at different time points post-infection (4–72 h p.i.) and analyzed by Western blotting to determine total cellular RPB1 levels.

**Figure 5:**
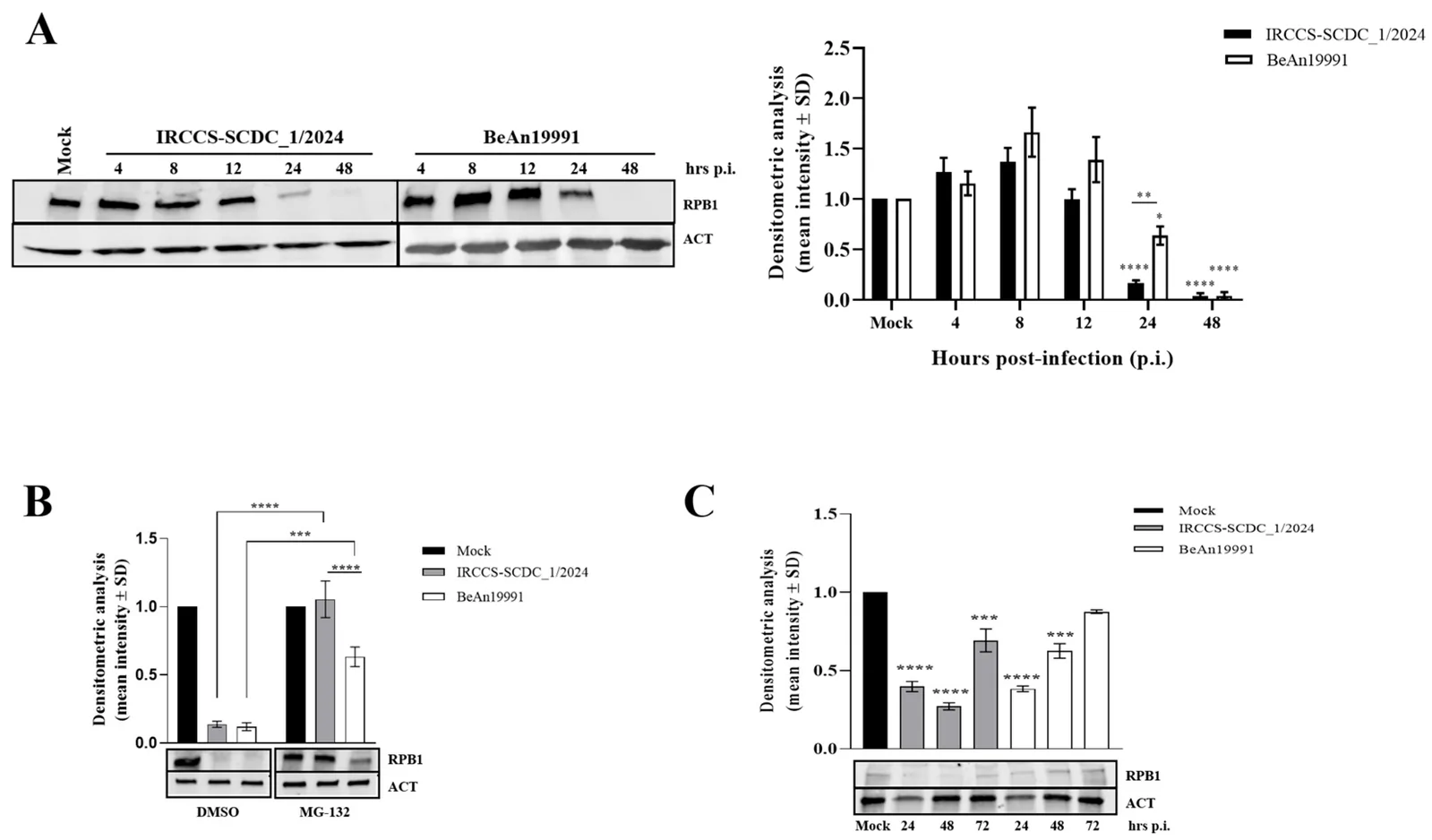
**Modulation of RPB1 subunits by OROV NSs in glioblastoma cells.** (**A**) DBTRG.05MG human glioblastoma cell line was infected with MOI = 0.1 of both BeAn19991 and IRCCS-SCDC_1/2024 OROV strains. Cell samples were collected at indicated time points post-infection (p.i.), WCLs were prepared, and 25 μg of total protein was resolved by SDS-PAGE. Cellular RPB1 content and ACT were probed by Western blotting with specific antibodies. A representative image of the Western blot is provided. Densitometric analysis was performed using ImageJ software, and values of the mean fold change in RPB1 band intensity ± standard deviations (SDs) were reported (right panel). Significance was evaluated by ANOVA test and reported as * *p* < 0.01, ** *p* < 0.001 and **** *p* < 0.0001 between infected or mock-infected samples. (**B**) The involvement of the proteasome in NSs-mediated RPB1 degradation was further investigated in DBTRG.05MG cells infected with indicated OROV strains and vehicle (DMSO) or MG-132-treated cells collected at 48 h p.i. Densitometric analysis was performed using ImageJ software, and values of the mean fold change in RPB1 band intensity ± SDs were reported (right panel). Significance was evaluated by ANOVA test and reported as *** *p* < 0.0005 and **** *p* < 0.0001 between infected or mock-infected samples. (**C**) Human primary fibroblast MRC-5 cells were infected with MOI = 0.1 of BeAn19991 or IRCCS-SCDC_1/2024 OROV strains. Cell samples were collected at the indicated time points p.i., and WCLs were prepared. Twenty-five μg of total protein was resolved by SDS-PAGE. Cellular RPB1 content and ACT were probed by Western blotting with specific antibodies. A representative image of the Western blot is provided. Densitometric analysis was performed using ImageJ software, and values of the mean fold change in RPB1 band intensity ± standard deviations (SDs) were reported (right panel). Significance was evaluated by ANOVA and reported as *** *p* < 0.001 and **** *p* < 0.0001 between infected or mock-infected samples.

A marked reduction in RPB1 abundance was observed at 24 h and up to 48 h p.i. for both viral strains, with IRCCS-SCDC_1/2024 causing a significantly greater reduction (86% reduction; *p* < 0.0001) than BeAn19991 (45% reduction; *p* = 0.01) (Figure 5A).

Overall, the IRCCS-SCDC_1/2024 strain displayed a more rapid and pronounced RPB1 depletion kinetic than the BeAn19991 strain, suggesting possible differences in pathogenic potential.

To assess whether NSs-mediated RPB1 degradation was proteasome-dependent in infected DBTRG.05MG cells, these were treated with the proteasome inhibitor MG-132 and collected at 48 h p.i., a time point at which RPB1 depletion was maximal following infection with both strains. MG-132 treatment partially restored RPB1 levels in a strain-dependent manner, supporting the hypothesis that OROV NSs promotes proteasome-mediated degradation of RPB1, although to different extents in the two viral strains (Figure 5B).

To further characterize this activity in a physiologically relevant target cell, we investigated RPB1 stability in primary human lung fibroblasts (MRC-5), which may represent one of the earliest sites of viral replication following transmission through a mosquito bite. In this model, both OROV strains induced an early reduction in RPB1 abundance, which was already evident at 24 h p.i. (Figure 5C) with the IRCCS-SCDC_1/2024 strain exhibiting a stronger and more sustained effect than the BeAn19991 reference strain. This activity persisted up to 72 h p.i., resulting in an approximately 30% reduction in RPB1 levels (*p* = 0.0008), whereas the effect of BeAn19991 was no longer statistically significant at the same time point (12% reduction; *p* = n.s.) (Figure 5C).

Despite these differences, a low and partial restoration of basal RPB1 levels was observed at later stages of infection for both strains, suggesting that the virus-induced transcriptional shutdown may be, at least in part, reversible over time (Figure 5C).

### 3.5. Ancestral Oropouche Virus Strain Displays Enhanced Replication Kinetics

**Figure 6:**
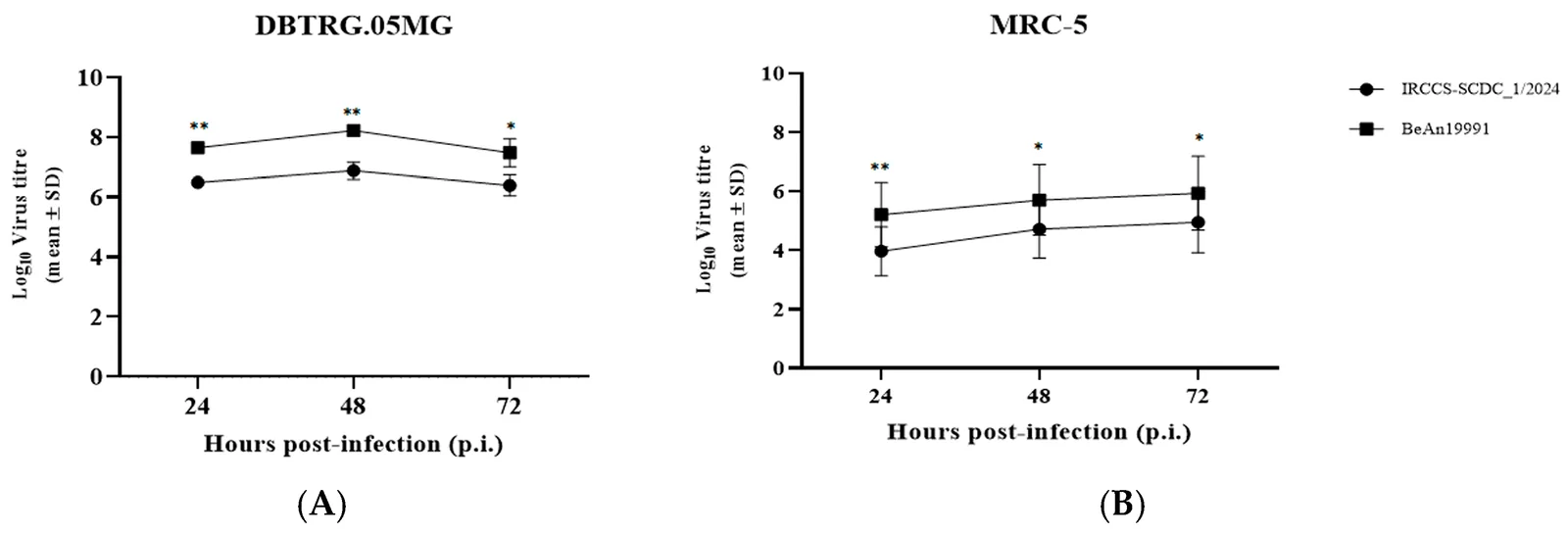
**Kinetic growth of BeAn19991 and IRCCS-SCDC_1/2024 OROV strains in different cell systems.** (**A**) DBTRG.05MG cells were infected with reference or reassortant OROV strains at an MOI of 0.1. Cell culture supernatants were collected at 24 h, 48 h, and 72 h p.i., and viable progeny virus content was assessed by a microtitration assay. (**B**) The kinetic growth of the above-described OROV strains was also investigated in primary fibroblast cells. Cell culture supernatants were collected at the indicated time points and assessed for virus replication by microtitration assay. Results were presented as Log_10_ of the mean viral titer expressed as Tissue Culture Infectious Dose 50%/mL (TCID_50_/mL) ± SD from at least three (*n* > 3) separate experiments. Significance was determined using an ANOVA test as * *p* < 0.05 and ** for *p* < 0.01.

Previous studies have shown that certain bunyaviruses exhibit different levels of fitness depending on the infected cell type, potentially due to differences in the generation of defective interfering particles [28,40,41,42]. To further characterize the biological properties of the selected OROV strains, viral replication was evaluated in both interferon-competent and interferon-responsive cellular systems at a multiplicity of infection (MOI) of 0.1. Since OROV is able to infect multiple cell types, including fibroblasts and neural cells [34,43], we examined the replication kinetics of the IRCCS-SCDC_1/2024 and BeAn19991 strains in DBTRG.05MG and MRC-5 cells to determine whether differences in viral growth could account for the distinct effects observed on IFN-β modulation and RNA polymerase II degradation. Viral replication was monitored in culture supernatants collected at 24, 48, and 72 h post-infection (p.i.), and infectious titers were determined by endpoint dilution assay (TCID_50_/mL). Replication analyses revealed a consistent growth disadvantage of the IRCCS-SCDC_1/2024 strain compared with the ancestral BeAn19991 strain in both cellular models and at all time points examined (Figure 6).

Overall, viral titers were higher in DBTRG.05MG cells than in MRC-5 fibroblasts. BeAn19991 maintained a clear replicative advantage over IRCCS-SCDC_1/2024, reaching peak titers at 48 h p.i. with a significant difference in viral production (mean log_10_ titer: 8.22 ± 0.10 vs. 6.87 ± 0.28, respectively; *p* = 0.004) (Figure 6).

These findings indicate that cell type strongly influences OROV replication kinetics and suggest that the higher levels of IFN-β produced in MRC-5 cells may contribute to restricting viral replication. Moreover, despite displaying more pronounced host shutoff activity, the contemporary IRCCS-SCDC_1/2024 strain exhibited reduced replicative fitness compared with the ancestral BeAn19991 strain, highlighting a potential trade-off between immune modulation and viral replication efficiency.

### 3.6. Oropouche Virus Strains Differentially MODULATE Type I Interferon Responses in a Time- and Cell-Dependent Manner

We next investigated the impact of the selected OROV strains on IFN-β production in DBTRG.05MG and MRC-5 cells by performing a time-course analysis.

**Figure 7:**
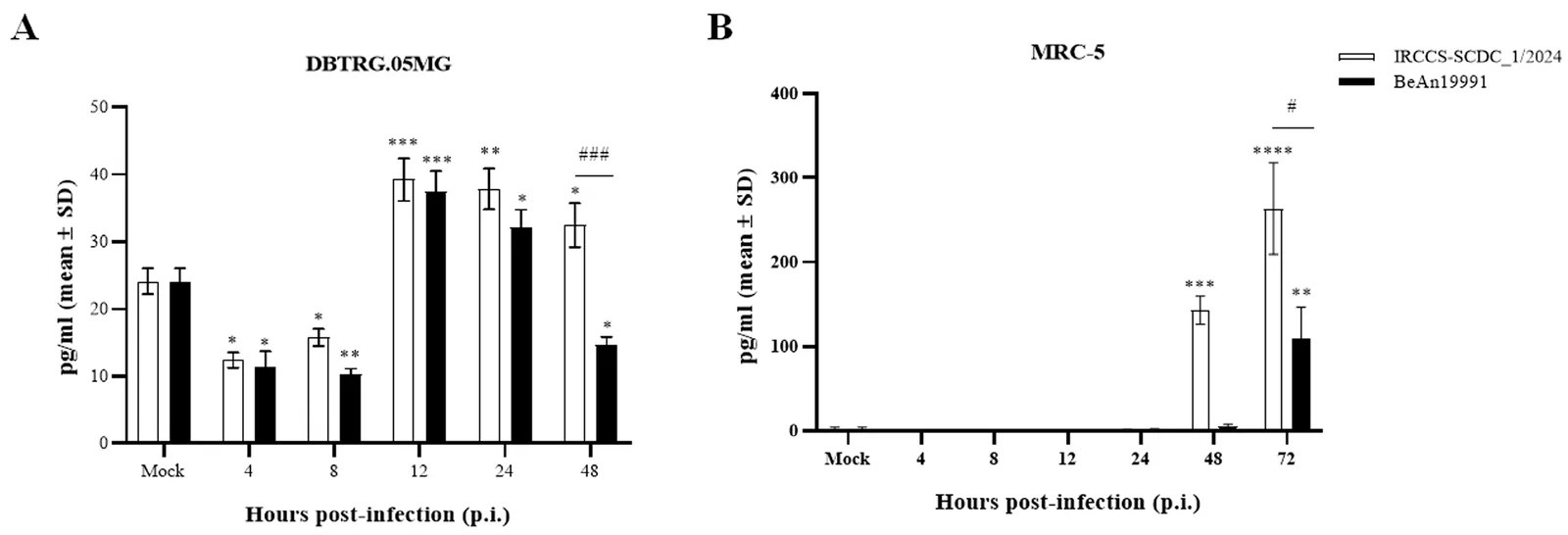
**Different modulation of IFN-β release by OROV strains.** Secreted IFN-β was assessed in (**A**) DBTRG-05MG and (**B**) MRC-5 cells infected (MOI = 0.1) by BeAn19991 or IRCCS-SCDC_1/2024 OROV strains by enzyme-linked immunoassay (ELISA) at indicated time points. Negative control (Mock) was represented by uninfected cells. Quantitative evaluation, based on relative standard curves, was performed, and results are reported as mean concentration (pg/mL) ± SD from at least three independent experiments (*n* ≥ 3). Significance was determined using an ANOVA test, where *p* values were listed as * *p* < 0.05, ** *p* < 0.01, *** *p* < 0.001, and **** *p* < 0.0001 with respect to the mock-infected control or # *p* < 0.05 and ### *p* < 0.001 between virus strains.

As shown in Figure 7A, infection of DBTRG.05MG cells with either IRCCS-SCDC_1/2024 or BeAn19991 at an MOI of 0.1 resulted in a marked suppression of IFN-β secretion during the early stages of infection (4–8 h p.i.).

As infection progressed, IFN-β levels gradually increased, although distinct patterns emerged between the two viral strains. In particular, BeAn19991-infected DBTRG.05MG cells displayed a decline in cytokine production at 48 h p.i. (14.69 ± 1.17 pg/mL) (Figure 7A), coinciding with peak viral replication and extensive virus-induced cytopathic effects (Figure 6). In contrast, MRC-5 fibroblasts exhibited delayed, but substantially stronger, IFN-β responses, with maximal cytokine production detected at 72 h p.i. (Figure 7B). Notably, despite its lower replication efficiency in MRC-5 cells (Figure 6), the IRCCS-SCDC_1/2024 strain induced significantly higher levels of IFN-β secretion than the BeAn19991 strain (263.5 ± 54.4 pg/mL; *p* = 0.01) (Figure 7B). Importantly, these differences could not be attributed to variations in virus-induced cell damage, as no evident cytopathic effect was observed in this cell system at the indicated time point (Figure 7B).

Consistent with previous reports [32], the ability of OROV to stimulate innate immune responses remained relatively limited when compared with other members of the Bunyavirales order, including several Orthobunyaviruses and phleboviruses. Collectively, these findings indicate that innate immune signaling is transiently suppressed during the early stages of OROV infection, even at low viral inocula. Furthermore, the differential modulation of IFN-β observed between the two OROV strains cannot be explained solely by NSs-mediated antagonism and likely involves additional viral determinants and/or host cell-specific factors that contribute to shaping the antiviral response.

## 4. Discussion

Previous in vitro studies using recombinant Oropouche virus (OROV) lacking the NSs gene demonstrated a critical role for NSs in suppressing type I interferon (IFN-α/β) induction and antagonizing the host innate immune response [29]. In light of the last OROV outbreaks across the Americas and the identification of imported OROV cases in Italy, we sought to further characterize the molecular mechanisms by which OROV NSs inhibits IFN-β expression and induces host cellular shutoff.

Previous studies have shown that members of the Retinoic Acid-Inducible Gene-I-Like Receptor (RLR) family, together with Mitochondrial Antiviral Signaling protein (MAVS) and the transcription factors Interferon Regulatory Factors 3 and 7 (IRF-3/7), play a central role in controlling OROV replication in vivo [33]. In parallel, recent evidence demonstrated that OROV NSs targets the RPB1 subunit of cellular RNA polymerase II, thereby suppressing host transcription [30]. However, it remained unclear whether the inhibition of type I interferon responses and the transcriptional shutoff activity mediated by NSs represented two independent functions or whether they were mechanistically linked. To address this question, we investigated the effects of NSs proteins derived from both the prototype BeAn19991 strain and the recently identified reassortant strain IRCCS-SCDC_1/2024 on key components of the IFN-β signaling pathway and on RPB1 function. Our results demonstrate that NSs protein from both viral strains efficiently inhibited IFN-β promoter activation induced by RIG-I, IRF-3, or the constitutively active IRF-3-5D mutant. These findings initially suggested that NSs interferes with multiple steps of the IFN-β signaling cascade. However, subsequent analyses revealed that the inhibitory activity of NSs was not restricted to innate immune signaling components. Indeed, the expression of an unrelated viral protein, the Toscana virus nucleoprotein (TOSV N), was similarly reduced in the presence of NSs. This broad inhibitory effect suggested that NSs do not selectively target specific antiviral signaling molecules but rather affect a fundamental cellular process required for gene expression. Notably, the reduction in protein accumulation was not associated with enhanced proteasomal degradation of the tested targets, as treatment with the proteasome inhibitor MG-132 failed to restore basal protein levels. In contrast, RT-qPCR analyses revealed a marked reduction in the abundance of all examined transcripts, including endogenous IFN-β, RIG-I, and ectopically expressed TOSV_N. These findings strongly support a model in which OROV NSs acts primarily at the transcriptional level. The role of OROV NSs as a transcriptional repressor through targeting of the RNA polymerase II large subunit RPB1 was recently described by Cobeña et al. [29]. Our data further extend these observations by demonstrating that all exogenous expression systems employed in this study, which rely on RNA polymerase II-dependent promoters, were similarly affected by NSs expression. Moreover, co-immunoprecipitation experiments clearly identified a direct interaction between OROV NSs and RPB1, providing additional evidence that host transcriptional machinery represents a major target of NSs activity. Consistent with this hypothesis, actinomycin D chase experiments showed that NSs expression did not alter the stability of the analyzed transcripts, indicating that the observed reduction in mRNA abundance results from impaired transcription rather than accelerated mRNA degradation.

Interestingly, no substantial differences were observed between the NSs proteins derived from the two OROV strains in overexpression experiments. These findings suggest that the ability to antagonize host transcription and innate immune responses represents a conserved function of OROV NSs that has been maintained throughout viral evolution.

While Cobeña et al. investigated NSs activity using a recombinant Rift Valley fever virus (RVFV) expressing the OROV NSs protein, our study examined the biological properties of two OROV isolates: the prototype Brazilian strain BeAn19991 and the reassortant strain IRCCS-SCDC_1/2024, recovered from the first imported OROV case identified in Europe. Furthermore, we employed two biologically relevant cellular models, namely human glioblastoma cells (DBTRG.05MG), providing an in vitro model of human brain-derived cells, and primary human fibroblasts (MRC-5), which may represent an early site of viral replication following mosquito transmission [3,6,10].

Replication studies revealed distinct growth characteristics between the two strains, with BeAn19991 consistently displaying higher replicative fitness than IRCCS-SCDC_1/2024 in both cell types. Despite this reduced replication efficiency, IRCCS-SCDC_1/2024 induced a more pronounced and rapid depletion of cellular RPB1, suggesting that the extent of host transcriptional shutoff does not directly correlate with viral replication kinetics. These findings raise the possibility that excessive host shutoff may impose a fitness cost on viral replication, although additional studies will be required to clarify this relationship.

Importantly, both OROV strains induced significant RPB1 degradation in glioblastoma and fibroblast cell models. In glia-derived cells, restoration of RPB1 levels following MG-132 treatment confirmed that proteasome-mediated degradation contributes to virus-induced transcriptional shutdown, extending previous observations obtained in heterologous experimental systems and demonstrating that this mechanism also occurs during infection with wild-type OROV strains. However, we acknowledge that DBTRG.05MG cells are a glioblastoma-derived cell line and may not fully recapitulate the physiological features of primary human neural cells, including innate immune signaling pathways. Therefore, our findings should be interpreted as evidence obtained in a human CNS-derived cellular model rather than as a direct demonstration of OROV behavior in neurons. Further studies using more physiologically relevant systems, such as primary human neurons, iPSC-derived neural cells, or brain organoids, as well as in vivo models, will be required to confirm whether NSs-mediated transcriptional suppression and RPB1 degradation also occur in the human CNS context. Nevertheless, recent findings by Connors et al. support the relevance of NSs-mediated mechanisms in neural systems, suggesting that similar processes may contribute to OROV neuroinvasion and pathogenesis [43]. As a consequence of the transcriptional shutdown induced by NSs, activation of the innate immune response was delayed during infection. Nevertheless, the two cellular models exhibited markedly different response profiles. In DBTRG.05MG cells, IFN-β production remained limited throughout infection, suggesting a weak antiviral response. In contrast, MRC-5 fibroblasts mounted a delayed but substantially stronger IFN-β response, particularly following infection with the IRCCS-SCDC_1/2024 strain. Despite this increased cytokine production, viral replication was not efficiently controlled during the early stages of infection, indicating that OROV-mediated suppression of host transcription likely provides a critical temporal window that allows productive viral replication before antiviral defenses become fully established.

Collectively, our findings support a model in which OROV NSs suppresses innate immune responses primarily through the inhibition of host RNA polymerase II activity and the consequent global shutdown of cellular transcription. Rather than selectively targeting individual components of the IFN-β signaling pathway, NSs appears to broadly impair host gene expression through its interaction with RPB1, thereby simultaneously preventing antiviral gene induction and promoting an intracellular environment favorable for viral replication. Furthermore, although this function is highly conserved between ancestral and contemporary OROV strains, differences in the magnitude of RPB1 depletion, IFN-β induction, and viral fitness suggest that additional viral determinants may contribute to strain-specific pathogenic properties and warrant further investigation.

## Data Availability

Dataset available on request from the authors.

## Supplementary Materials

The following supporting information can be downloaded at: https://www.mdpi.com/article/10.3390/v18080828/s1, Figure S1: **BeAn19991 and IRCCS-SCDC_1/2024 NSs comparison**.

## Abbreviations

The following abbreviations are used in this manuscript:

RPB1	RNA polymerase II subunit B1
OROV	Oropouche virus
NSs	non-structural protein S
IFN-β	interferon-beta
RIG-I	Retinoic Acid-Inducible Gene I
IRF-3	Interferon Regulatory Factor-3
