# Supplementary material for: Oropouche Virus: Comparative Study of Two Strains Reveals Distinct Innate Immunity Modulation
Source: Viruses. 2026 Jul 28;18(8):828. doi: 10.3390/v18080828 (PMC13517354; doi:10.3390/v18080828)
Supplement: Supplementary file 1 [file viruses-18-00828-s001.zip › viruses-4400002-supplementary.pdf]

```
BeAn19991           MYHNGLHLHLIRRQHMWHLKLDTDKCSMLVLLESSSSTKRRPKMSYVRHRGPWLTLLLVG
IRCCS-SCDC_1/2024   MYHNGLHLHLIRRQHMWHLKLDTDKCSMLVLLESSSSTKRRPKMSYVRHRGPWLTLLLVG
                    ************************************************************

BeAn19991           SNLQWLITISHSSSRIQCRTTVLPCTVCQDT
IRCCS-SCDC_1/2024   SNLQWLTTISHNSSRIQCRTTVLPCTVCQDT
                    ****** ****.*******************
```

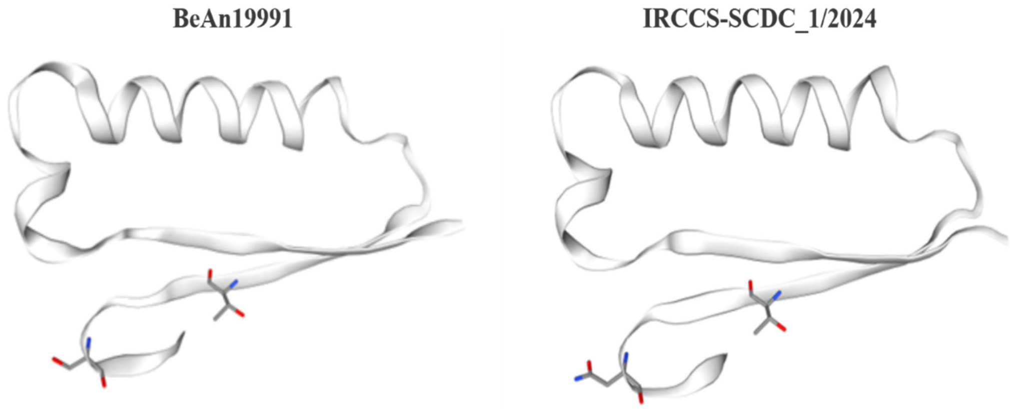

Figure S1. BeAn19991 and IRCCS-SCDC_1/2024 NSs comparison. The homology between NSs protein from the two OROV strains has been evaluated by sequence alignment, highlighting the presence of I67T and S72N mutations between the reference and the reassortant strain. Protein structure prediction was assessed by SWISS-MODEL (https://swissmodel.expasy.org/) online tool.
